# Antioxidant supplements for prevention of mortality in healthy participants and patients with various diseases

**DOI:** 10.1590/1516-3180.20151332T1

**Published:** 2014-11-28

**Authors:** Goran Bjelakovic, Dimitrinka Nikolova, Lise Lotte Gluud, Rosa G. Simonetti, Christian Gluud

## Abstract

**BACKGROUND:**

Our previous systematic review has demonstrated that antioxidant supplements may increase mortality. We have now updated this review.

**OBJECTIVES:**

To assess the beneficial and harmful effects of antioxidant supplements for prevention of mortality in adults.

**METHODS:**

*Search methods:* We searched The Cochrane Library, Medline, Embase, Lilacs, the Science Citation Index Expanded, and Conference Proceedings Citation Index-Science to February 2011. We scanned bibliographies of relevant publications and asked pharmaceutical companies for additional trials.

*Selection criteria:* We included all primary and secondary prevention randomized clinical trials on antioxidant supplements (beta-carotene, vitamin A, vitamin C, vitamin E, and selenium) versus placebo or no intervention.

*Data collection and analysis:* Three authors extracted data. Random-effects and fixed-effect model meta-analyses were conducted. Risk of bias was considered in order to minimize the risk of systematic errors. Trial sequential analyses were conducted to minimize the risk of random errors. Random effects model meta-regression analyses were performed to assess sources of intertrial heterogeneity.

**MAIN RESULTS:**

Seventy-eight randomized trials with 296,707 participants were included. Fifty-six trials including 244,056 participants had low risk of bias. Twenty-six trials included 215,900 healthy participants. Fifty-two trials included 80,807 participants with various diseases in a stable phase. The mean age was 63 years (range 18 to 103 years). The mean proportion of women was 46%. Of the 78 trials, 46 used the parallel-group design, 30 the factorial design, and 2 the cross-over design. All antioxidants were administered orally, either alone or in combination with vitamins, minerals, or other interventions. The duration of supplementation varied from 28 days to 12 years (mean duration 3 years; median duration 2 years). Overall, the antioxidant supplements had no significant effect on mortality in a random-effects model meta-analysis (21,484 dead/183,749 (11.7%) versus 11,479 dead/112,958 (10.2%); 78 trials, relative risk (RR) 1.02, 95% confidence interval (CI) 0.98 to 1.05) but significantly increased mortality in a fixed-effect model (RR 1.03, 95% CI 1.01 to 1.05). Heterogeneity was low with an I2- of 12%. In meta-regression analysis, the risk of bias and type of antioxidant supplement were the only significant predictors of intertribal heterogeneity. Meta-regression analysis did not find a significant difference in the estimated intervention effect in the primary prevention and the secondary prevention trials. In the 56 trials with a low risk of bias, the antioxidant supplements significantly increased mortality (18,833 dead/146,320 (12.9%) versus 10,320 dead/97,736 (10.6%); RR 1.04, 95% CI 1.01 to 1.07). This effect was confirmed by trial sequential analysis. Excluding factorial trials with potential confounding showed that 38 trials with low risk of bias demonstrated a significant increase in mortality (2822 dead/26,903 (10.5%) versus 2473 dead/26,052 (9.5%); RR 1.10, 95% CI 1.05 to 1.15). In trials with low risk of bias, beta-carotene (13,202 dead/96,003 (13.8%) versus 8556 dead/ 77,003 (11.1%); 26 trials, RR 1.05, 95% CI 1.01 to 1.09) and vitamin E (11,689 dead/97,523 (12.0%) versus 7561 dead/73,721 (10.3%); 46 trials, RR 1.03, 95% CI 1.00 to 1.05) significantly increased mortality, whereas vitamin A (3444 dead/24,596 (14.0%) versus 2249 dead/16,548 (13.6%); 12 trials, RR 1.07, 95% CI 0.97 to 1.18), vitamin C (3637 dead/36,659 (9.9%) versus 2717 dead/ 29,283 (9.3%); 29 trials, RR 1.02, 95% CI 0.98 to 1.07), and selenium (2670 dead/39,779 (6.7%) versus 1468 dead/22,961 (6.4%); 17 trials, RR 0.97, 95% CI 0.91 to 1.03) did not significantly affect mortality. In univariate meta-regression analysis, the dose of vitamin A was significantly associated with increased mortality (RR 1.0006, 95% CI 1.0002 to 1.001, P = 0.002).

**AUTHORS’ CONCLUSIONS:**

We found no evidence to support antioxidant supplements for primary or secondary prevention. Beta-carotene and vitamin E seem to increase mortality, and so may higher doses of vitamin A. Antioxidant supplements need to be considered as medicinal products and should undergo sufficient evaluation before marketing.

This is the abstract of a Cochrane Review published in the Cochrane Database of Systematic Reviews (CDSR) 2012, issue 3. Art. No.: CD007176. DOI: 10.1002/14651858.CD007176.pub2. For full citation and authors’ details see reference 1.

The abstract of this review is available from: http://onlinelibrary.wiley.com/doi/10.1002/14651858.CD007176.pub2/abstract.
